# 4475 Meeting Partners Where They Are: Tailoring Community-Engaged Research Consultation Services

**DOI:** 10.1017/cts.2020.275

**Published:** 2020-07-29

**Authors:** Adam Paberzs, Patricia Piechowski, Jordan Poll, Meghan Spiroff, Karen Calhoun, Ayse Buyuktur, Athena McKay, Donald Vereen, Susan Woolford

**Affiliations:** 1 University of Michigan School of Medicine; 2 Michigan Institute for Clinical and Health Research; 3 Community-Based Public Health, University of Michigan; 4 University of Michigan, Pediatrics

## Abstract

OBJECTIVES/GOALS: One of the most significant challenges to community engagement experienced by Clinical and Translational Science Award (CTSA) institutions is inadequate capacity of academic and community partners to engage in collaborative research. Several CTSAs within the consortium provide consultation services to help address this gap. METHODS/STUDY POPULATION: For over 10 years, the Michigan Institute for Clinical and Health Research (MICHR), a CTSA at the University of Michigan, has provided CEnR-specific consultations to partners seeking support for a variety of needs. Consultations can be requested for assistance with identifying potential partners, developing partnership infrastructure, finding CEnR funding opportunities, and incorporating CEnR approaches into research plans. When a consultation is requested, MICHR’s Community Engagement (CE) Program responds by planning a meeting with staff and faculty who have relevant skills, expertise, and connections. After the initial meeting, the CE Program provides follow-up communication and support based on the needs of the specific request, and often facilitates connections with potential partners. RESULTS/ANTICIPATED RESULTS: The two most frequent types of consultation requests involve 1) making connections with potential researchers or community partner organizations, and 2) providing guidance on research grant applications that involve community engagement. MICHR provides approximately 50 CEnR consultations each year, which have resulted in development of new partnerships, grant submissions, and research projects that utilize CEnR principles and address community-identified health priorities. DISCUSSION/SIGNIFICANCE OF IMPACT: This presentation will describe the evolution of MICHR’s CEnR consultation process and highlight successful outcomes and lessons learned over its 12-year history. CONFLICT OF INTEREST DESCRIPTION: NA

